# Unveiling solutions: strategies for tuberculosis control and diagnosis in the Dominican Republic and La Hispaniola

**DOI:** 10.1186/s44263-024-00052-7

**Published:** 2024-03-21

**Authors:** Robert Paulino-Ramírez, Rita Rojas, Maridania Jabier

**Affiliations:** 1 Instituto de Medicina Tropical & Salud Global, Universidad Iberoamericana (UNIBE), UNIBE Research Hub, Santo Domingo, 22333 Dominican Republic; 2 Infectious Disease Department, Hospital General Plaza de La Salud, Santo Domingo, Dominican Republic; 3 Bionuclear, Laboratory Development Department, Santo Domingo, Dominican Republic

Tuberculosis (TB) is a global health issue, especially affecting countries in the Global South, including the Dominican Republic. This comment discusses TB epidemiology, HIV co-infection and stigma-associated intersectionality, and diagnosis barriers, offering insights for future elimination strategies in the country and beyond.

## Tuberculosis in the global context

Prior to the onset of the COVID-19 pandemic, tuberculosis (TB) held the position of being the predominant infectious disease, surpassing HIV/AIDS in prevalence. Today, TB remains a significant global health challenge, with millions of new cases reported each year. In 2022, an estimated 10.6 million people fell ill with TB globally and preventing the rapid transmission of multi-drug resistant TB (MDR-TB) strains remains as one of the many concerns for TB control programs across the globe [1].

The history of TB in Hispaniola, the island that comprises the modern-day countries of Haiti and the Dominican Republic (DR), is complex and intertwined with the broader history of the Caribbean region. It is known that indigenous populations faced various health challenges, including infectious diseases. With the arrival of Spaniards in 1492, followed by European colonization, new diseases, including TB, were introduced to the indigenous populations. The lack of immunity to these diseases largely contributed to the devastating impact on the native communities [2].

In the Americas, 325,000 new TB cases were estimated in 2022, but some researchers consider those numbers are largely underreported. This represents a 4% increased incidence compared with 2021 [3]. The utilization of rapid tests recommended by the World Health Organization (WHO) for diagnosis has risen from 25% in 2019 to 40% in 2022, although it remains considerably below the recommended threshold of 90% for the Ending TB Strategy [4]. While the coverage of drug sensitivity tests has expanded, enabling the identification of drug-resistant TB strains, universal access remains elusive, posing a challenge in the diagnosis of drug-resistant TB. Furthermore, coverage for rifampicin sensitivity tests has experienced an increase from 44% in 2019 to 59% in 2022. Similarly, for second-line drugs such as fluoroquinolones, testing has surged from 18% in 2019 to 50% in 2022 [1]. Despite some fluctuations, treatment outcomes have not undergone substantial changes in recent years, signifying missed opportunities, instances of mortality, or individuals lost to follow-up. When the TB care cascade is analyzed, diagnosis remains the weakest link in it [4]. Among individuals with drug-sensitive TB, 72% achieved cure after adequate treatment. In contrast, for TB/HIV coinfection, the cure rate was only 53%, and for cases with MDR-TB, and extensively drug-resistant TB (XDR-TB), the cure rate was 60%, and 57% respectively [1].

In high TB burden settings, the current approach to TB detection relies on passive case finding, where patients must recognize their illness, seek timely care, and health services must respond efficiently. Unfortunately, symptomatic patients often take up to three months to present to the health sector, leading to an additional month for TB diagnosis [5]. Health care provision capacity varies significantly both between and within countries, resulting in many symptomatic patients not being diagnosed due to a lack of appropriate testing [6]. Conducting patient-pathway analyses could help align services with patient needs, improving the design of person-centered programs and addressing some of these deficiencies [5, 6]. Community-wide screening has shown effectiveness in reducing TB prevalence and infection rates in many settings. However, the replication of these results in scaled-up, programmatic situations require further implementation and health system research. While targeting high-risk groups like people with HIV (PWH) and household contacts (Index testing) may not identify all TB cases, it remains beneficial for those detected and offered treatment, especially during the transition from high to low prevalence [7].

Over the past decade, molecular technology has replaced sputum smear microscopy as the primary diagnostic test at health facilities, with the Xpert MTB/RIF assay being the WHO-preferred test due to its minimal expertise requirement, short processing time, high sensitivity and specificity, and ability to detect rifampicin resistance [8]. However, the practical challenges such as unstable electricity, poor training, maintenance issues, out-of- pocket payment, and interrupted cartridge supplies hinder the effective use of molecular diagnostic technology in the field [8]. There remains an unmet need for a highly sensitive and specific test, not necessarily sputum-based, deployable at the lowest level of the healthcare system.

## TB in the DR

Despite many efforts, in the DR, more than 4000 TB cases were reported in 2022, of these 77% diagnoses were made using rapid tests platforms (e.g., Xpert MTB/RIF). However, only 84% of these diagnoses received TB treatment, and 12% were lost to follow-up. Among PWH and TB, treatment success was lower (70%), and 12% died [1]. Some researchers suggest that although diagnostic systems have been improved, there are barriers to accessing health services. Among these factors, the return of TB results, the intersectionality of the stigma associated with the disease, which borders on the stigma associated with HIV, immigration status, socio-economic factors, among others, play a significant role in TB control [9]. As TB is often regarded as an indicator of HIV positivity, leading to the transfer of HIV-related stigma to individuals with TB, in countries with high burden of HIV associated stigma like the DR [10], it does not matter how good diagnostic systems are if healthcare environments present a barrier to users. Some HIV stigma reduction programs in health providers have shown that they could improve this reality and ensure retention and access to medications in a timely manner [11].

In contrast, although the diagnosis and treatment of active TB is the priority in its control, the identification and treatment of latent infections represent a great health concern because any latent infection can become active in any moment. For this reason, TB preventive treatment (TPT) is one of the prioritized programmatic strategies in tropical environments. Studies indicate that TPT proves beneficial in countries like the DR and Haiti, demonstrating a reduction in active TB incidence among high-risk populations [3]. Nevertheless, the financial strain associated with TB diagnosis and treatment poses barriers for patients and households, underscoring the necessity for enhanced access to care. While previous work also points to historical conflicts on Hispaniola significantly affecting the scope and early identification of cases of latent infection [9], further research into the factors that affect access to health care and retention is needed.

## Strategies to address TB in the DR

It is crucial to recognize the unique hurdles faced by the Global South in addressing the challenges associated with TB. Countries such as the DR often contend with limited healthcare infrastructure—including the absence of the first levels of care, scarce resources, and socioeconomic disparities, including stigma. Achieving the ambitious goal of TB elimination requires a comprehensive approach, with a key emphasis on timely and accurate diagnosis. Early detection of TB is paramount in preventing its spread and improving treatment outcomes. TB elimination necessitates interrupting transmission chains, and accurate diagnosis enables healthcare systems to identify and isolate infectious individuals promptly. This, in turn, prevents further spread of TB within households, communities, and healthcare settings.

Despite the global recognition of the significance of addressing TB-stigma, there is a shortage of high-quality studies assessing interventions aimed at diminishing TB-stigma. Some innovative conceptual frameworks evaluated previously in the DR emphasize that individuals with TB may encounter internalized stigma, as well as anticipated or enacted stigma from three distinct groups: the public, TB healthcare workers, and other healthcare workers (HCWs) [11]. Interventions targeting individuals with TB can effectively offer psychosocial support, contributing to treatment completion. Actions directed at HCWs can bolster the assistance provided to individuals with TB, while interventions targeting the public hold the potential to diminish stigma at the community level. The most effective and synergistic intervention would involve targeting more than one key population.

## Conclusions

Evidence suggests that there is a need for tailored TB strategies that account for the challenges faced by regions most affected by the disease, such as introducing decentralized healthcare delivery, community-based interventions, and sustainable financing mechanisms. Timely TB diagnosis is pivotal for patient outcomes, transmission control, and curbing drug resistance. A comprehensive approach aimed at integrating diagnostics, treatment, and prevention is vital for global TB elimination. Continued research and innovation are crucial to enhance diagnostic tools, especially molecular assays, and point-of-care tests. Addressing TB stigma requires interventions targeting individuals, HCWs, and the public. Multi-pronged strategies are most effective. Incorporating stigma reduction in national and international TB strategies, including binational collaborations, is essential for developing and evaluating stigma-reducing interventions.

Despite advancements in medical science, achieving meaningful progress in TB elimination demands a holistic approach that addresses the complex interplay of social, economic, and health factors prevalent in these regions.

## Data Availability

No datasets were generated or analysed during the current study.
